# Cabbage looper (*Trichoplusia ni* Hübner) labial glands contain unique bacterial flora in contrast with their alimentary canal, mandibular glands, and Malpighian tubules

**DOI:** 10.1002/mbo3.994

**Published:** 2020-01-28

**Authors:** Susan D. Lawrence, Nicole G. Novak, Jonathan Shao, Saikat Kumar B. Ghosh, Michael B. Blackburn

**Affiliations:** ^1^ Invasive Insect Biocontrol and Behavior Lab USDA‐ARS Beltsville Maryland; ^2^ North East Area, USDA‐ARS Beltsville Maryland; ^3^ School of Medicine Center for Inflammatory and Vascular Diseases University of Maryland Baltimore Maryland

**Keywords:** cabbage looper, gut, labial glands, microbiome, plant–herbivore interactions, *Trichoplusia ni*

## Abstract

In recent years, several studies have examined the gut microbiome of lepidopteran larvae and how factors such as host plant affect it, and in turn, how gut bacteria affect host plant responses to herbivory. In addition, other studies have detailed how secretions of the labial (salivary) glands can alter host plant defense responses. We examined the gut microbiome of the cabbage looper (*Trichoplusia ni*) feeding on collards (*Brassica oleracea*) and separately analyzed the microbiomes of various organs that open directly into the alimentary canal, including the labial glands, mandibular glands, and the Malpighian tubules. In this study, the gut microbiome of *T. ni* was found to be generally consistent with those of other lepidopteran larvae in prior studies. The greatest diversity of bacteria appeared in the Firmicutes, Actinobacteria, Proteobacteria, and Bacteriodetes. Well‐represented genera included *Staphylococcus*, *Streptococcus*, *Corynebacterium*, *Pseudomonas*, *Diaphorobacter*, *Methylobacterium*, *Flavobacterium*, and *Cloacibacterium*. Across all organs, two amplicon sequence variants (ASVs) associated with the genera *Diaphorobacter* and *Cloacibacterium* appeared to be most abundant. In terms of the most prevalent ASVs, the alimentary canal, Malpighian tubules, and mandibular glands appeared to have similar complements of bacteria, with relatively few significant differences evident. However, aside from the *Diaphorobacter* and *Cloacibacterium* ASVs common to all the organs, the labial glands appeared to possess a distinctive complement of bacteria which was absent or poorly represented in the other organs. Among these were representatives of the *Pseudomonas*, *Flavobacterium*, *Caulobacterium*, *Anaerococcus*, and *Methylobacterium*. These results suggest that the labial glands present bacteria with different selective pressures than those occurring in the mandibular gland, Malpighian tubules and the alimentary canal. Given the documented effects that labial gland secretions and the gut microbiome can exert on host plant defenses, the effects exerted by the bacteria inhabiting the labial glands themselves deserve further study.

## Graphical abstract

Labial gland secretions and the gut microbiota of insects affect defense responses of plants to herbivory. However, the microbiome of the labial gland has not been investigated. This work using cabbage looper, a serious pest of cole crops, demonstrates that the labial glands possess a community of bacteria that is distinct from the gut and other associated organs. This discovery warrants additional research to determine what role the labial gland microbiota has on the plant response to insect pests.

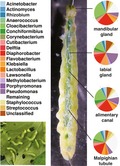

## 1 INTRODUCTION

Cabbage looper (*Trichoplusia ni*) is a voracious insect pest, which can eat ~160 different plant hosts. It prefers cruciferous species such as *Brassica oleracea*, hence the name cabbage looper. The plant response to insect infestation depends on the mode of infestation. Piercing/sucking insects with haustellate mouthparts do not create much damage upon infestation but generally induce the salicylic acid pathway and the defense genes involved in slowing plant pathogens. Chewing insects with mandibulate mouthparts typically cause the induction of the jasmonic acid pathway, which produces a cascade of defense genes that can deter insects via toxicity or slowing down digestive processes (Stahl, Hilfiker, & Reymond, 2018). However, plant responses to an insect pest are driven by more than just the mechanical damage caused by insect mouthparts. Plant responses to herbivory are altered by exposure to components of oral secretions from the labial (salivary) and mandibular glands, regurgitant from the alimentary canal, and frass. Additionally, microbes associated with the insect have been shown to alter how plants perceive herbivory. Generally, both chemical components within the oral secretions, as well as specific microbes isolated from the insect pest, can change the plant response (Stahl et al., 2018).

In recent years, the mechanisms that plants use to defend themselves against lepidopteran larvae, and the mechanisms the larvae employ to defeat those defenses, have been the subject of numerous investigations. Increasingly, these studies suggest that the insect–plant interaction can be substantially influenced or mediated by bacteria inhabiting the alimentary canal of the insect. In some instances, the resident bacteria favor the insect, while in other cases the plant benefits. Studies indicate that bacterial populations within two insect species inhabiting the same plant, or a single insect species on different plants can be quite variable, and appear to be highly influenced by the bacteria inhabiting the host plant (Jones, Mason, Felton, & Hoover, 2019).

One of the more straightforward ways gut bacteria can influence the interaction between an insect and its host plant is to augment the digestive processes of the insect to overcome factors that impair digestion. Multiple lines of evidence suggest that proteases produced by the gut bacteria of the velvetbean caterpillar allow the larvae to overcome the protease inhibitors produced by soybean (Visôtto, Oliveira, Guedes, Ribon, & Good‐God, 2009; Visôtto, Oliveira, Ribon, Mares‐Guia, & Guedes, 2009). In other insect–plant combinations, such as fall armyworm feeding on maize, it appears that gut bacteria may augment certain plant defenses to the detriment of the insect (Mason et al., 2019). Acevedo et al. (2017) demonstrated that a number of bacteria of the Enterobacteriaceae isolated from fall armyworm modulated jasmonic acid‐mediated plant defenses in a plant dependent way. *Pantoea ananatis* and an isolate termed Enterobacteriaceae‐1 downregulated polyphenol oxidase and a trypsin protease inhibitor in tomato, but upregulated a maize proteinase inhibitor in maize.

In addition to the advances in understanding the role of the gut microbiome in plant–caterpillar interactions, the role of the larval labial gland has also been examined using proteomic and transcriptomic methods (de la Paz Celorio‐Mancera et al., 2011; Rivera‐Vega, Acevedo, & Felton, 2017; Rivera‐Vega, Galbraith, Grozinger, & Felton, 2017; Rivera‐Vega, Stanley, Stanley, & Felton, 2018) Labial gland secretions from the cotton bollworm, *Helicoverpa armigera*, included a variety of enzymes with apparent digestive functions, such as proteases, lipases, and amylases. A variety of antimicrobial peptides and lysozymes were also detected, as was glucose oxidase (GOX), which decreases wound‐inducible nicotine production in tobacco (Musser et al., 2005; de la Paz Celorio‐Mancera et al., 2011). Transcriptome and proteome profiles of *Trichoplusia ni* labial glands were significantly different when the larvae were fed either tomato, considered a more challenging host, or cabbage. A variety of proteins involved in digestion and response to host defenses were upregulated when larvae were fed tomato relative to cabbage. In particular, the levels of catalase, which inhibits foliar peroxidase by reducing levels of H_2_O_2_, were found to be increased in the labial glands of larvae fed tomato (Rivera‐Vega, Galbraith, et al., 2017; Rivera‐Vega et al., 2018).

The labial glands, as well as the mandibular glands, also open directly into the oral cavity of the alimentary canal (Eaton, 1988). The alimentary canal of lepidopteran larvae is a tube of epithelium which runs the entire length of the larvae, from the oral cavity to the anus. It can be subdivided into three major functional regions, the foregut, midgut, and the hindgut. The foregut is a relatively narrow, muscular tube that conveys chewed leaf material from the oral cavity through the head and thorax to the midgut, which begins near the junction of the thorax and abdomen, occupying most of the latter. The midgut is the primary region responsible for secretion of digestive enzymes and absorption of nutrients and is composed of a thicker, columnar epithelium. The hindgut is the final segment of the alimentary canal and is involved and crucial in regulating hydration and electrolyte balance of the larvae. The alimentary canal includes several significant accessory structures that open directly into its lumen. These include labial and mandibular glands and the Malpighian tubules, which are the major excretory organs of the larvae. The Malpighian tubules are long, convoluted tubes which are closed at their distal ends and open into the hindgut just posterior to its junction with the midgut.

In the current study, we examined the alimentary canal microbiome of *T. ni*, including accessory structures that communicate directly with the gut lumen: the labial glands, mandibular glands, and the Malpighian tubules. We were particularly interested in determining whether the insect labial glands contained populations of bacteria, and if so, whether these populations were similar to those inhabiting the alimentary canal. To initiate the study of interactions between *T. ni* and *Brassica oleracea*, the bacteria associated with *T. ni* labial glands, mandibular labial glands, the alimentary canal, and the Malpighian tubules were characterized using next‐generation sequencing of 16S ribosomal RNA gene amplicons.

## 2 MATERIALS AND METHODS

### 2.1 Plant/insect preparation

Two flats of Champion collard seeds (Johnny's Selected Seeds) were sown in sunshine mix #1 (Griffin) and grown for 3 weeks. One flat of collards were moved to a greenhouse where the temperature mimicked the outdoor temperatures.

Four to six squares of wax paper with approximately 100 eggs each of recently oviposited *T. ni* eggs (Benzon Research) were pinned to the leaves throughout the flat that had been placed inside a mesh cage. The second flat of collards was added to the cage when larvae consumed ¾ of the first flat. Larvae were monitored until they reached the 5th instar and were collected for dissection.

### 2.2 Larval dissection

**Figure 1:**
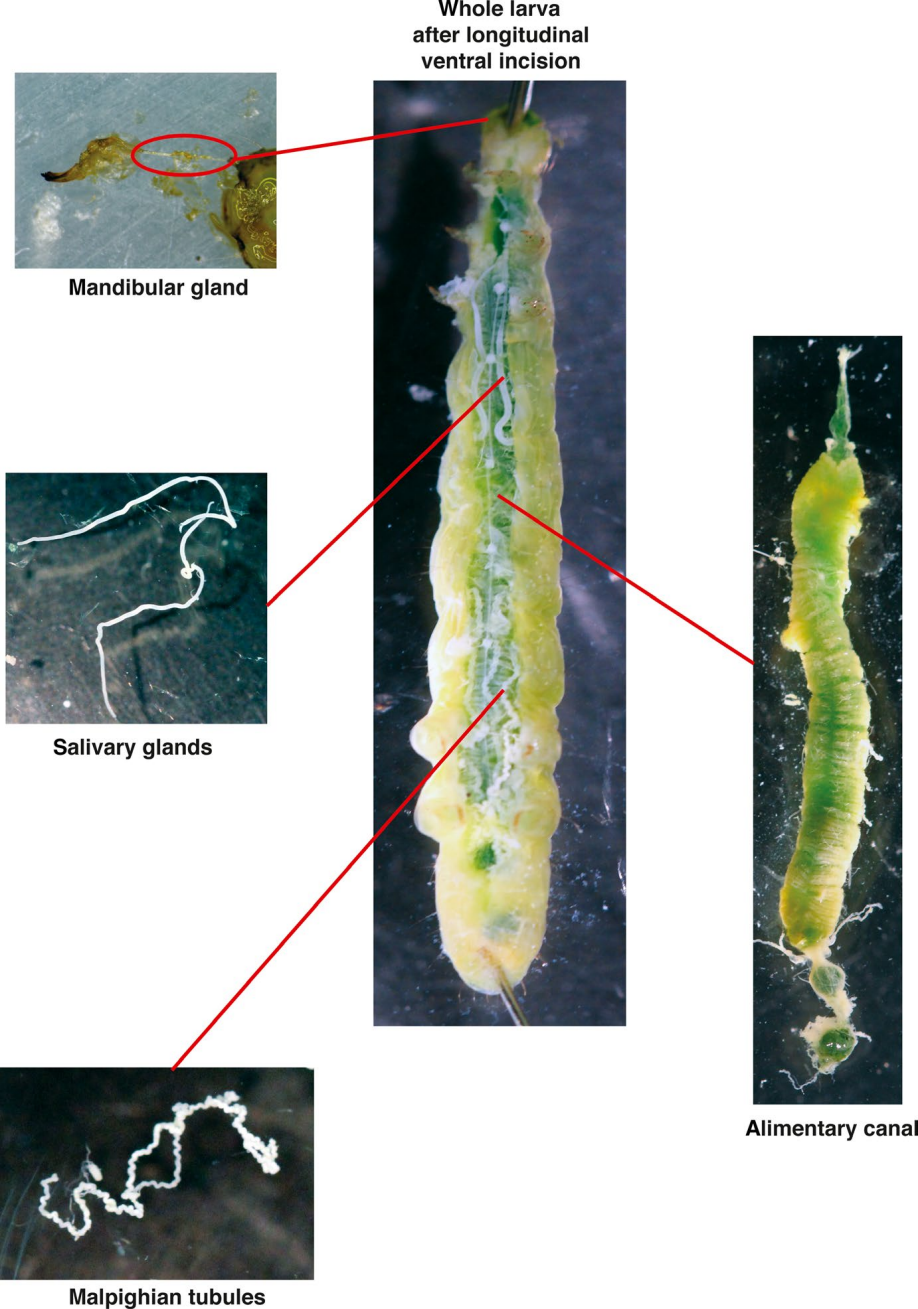
Organs used in current study

Larvae were sedated by placing at −20°C for 1–2 min to facilitate immobilization before dissection of labial glands, mandibular glands, Malpighian tubules, and midgut tissue (Figure 1). Five to ten organs (or sets of organs) were pooled for each biological replicate and frozen. Midgut tissue was rinsed in PBS (phosphate‐buffered saline) before freezing. Tissue was stored at −80°C until genomic DNA extraction.

### 2.3 Bacterial genomic DNA extraction and purification

Both GeneJET Genomic DNA purification (Thermo Scientific) and Gentra Puregene (Qiagen) kit reagents were used to isolate microbial genomic DNA to ensure liberation of all bacterial cells from insect organ tissue and efficient lysis of both gram‐negative and gram‐positive bacterial cells. Insect tissue samples stored at −80°C in microcentrifuge tubes were thawed on ice and ground with sterile mini pestles until a homogeneous mixture was achieved.

#### 2.3.1 GeneJET protocol

Immediately following tissue disruption, 9 μl GeneJET digestion solution per mg of tissue was added and samples were incubated at 56°C for 3 hr on a thermomixer (150 rpm every 10 min) until all particulates disappeared. The solution was centrifuged for 5 min at 16,000 × *g* to pellet any unlysed gram‐positive or gram‐negative bacterial cells (pellets were placed on ice for gram‐positive bacterial genomic DNA isolation using Gentra Puregene). The supernatant was transferred to new microcentrifuge tubes and further processed for gram‐negative bacterial DNA purification with GeneJET.

To continue with gram‐negative bacterial DNA purification, 20 μl of GeneJET RNase A solution was added to the supernatant, mixed by inversion and incubated for 10 min at room temperature. 200 μl of GeneJET lysis solution was added and mixed by inversion until a homogenous mixture was obtained. 400 μl of 50% ethanol was added and mixed by inversion followed by GeneJET column purification as per manufacturer instructions.

#### 2.3.2 Gentra Puregene protocol

The pellet from the initial centrifugation step in the GeneJET protocol was processed for gram‐positive bacterial cells as follows. 300 μl of Gentra Puregene cell suspension solution was added to the pellet and heated to 95°C for 10 min, then cooled to 37°C. 3 μl Gentra Puregene lytic enzyme solution was added, mixed by inversion 25 times, incubated for 30 min at 37°C, and finally centrifuged 1 min 16,000 × *g* to pellet cells. The supernatant was discarded, 300 μl Gentra Puregene cell lysis solution was added, and the remaining pellet was gently resuspended by flicking the tube. Resuspended pellet solutions were incubated at 80°C for 5 min to complete gram‐positive cell lysis. 1.5 μl of Gentra Puregene RNase A solution was added, and tubes were mixed by inversion 25 times then incubated for 1 hr at 37°C followed immediate cooling on ice for 1 min. 100 μl Gentra Puregene protein precipitation solution was added, vortexed for 20 s then centrifuged 16,000 × *g* for 3–8 min until a tight protein pellet formed. The supernatant was transferred to a new 1.5 ml microcentrifuge tube containing 300 μl isopropanol and inverted gently 50 times. The DNA was pelleted by centrifugation at 16,000 × *g* for 1 min, washed with 70% ethanol and air‐dried for 5 min at room temperature. 100 μl Gentra Puregene DNA Hydration Solution was added, and the DNA was incubated at 65°C for 1 hr to aid in dissolving the DNA. Purified genomic DNA samples from both GeneJET and Gentra workflows for each replicate were combined prior to DNA quality assessment.

### 2.4 DNA quality assessment and concentration measurements

Before library preparation, DNA quality was assessed. First, concentrations of the DNA samples were determined with a plate fluorometer. To begin library preparation, 30 µl of 25 ng/µl DNA was necessary to build Illumina compatible 16S libraries (Illumina, 2013). The concentrations of the samples that met these criteria, and a random selection of samples were analyzed using a Fragment Analyzer to ensure reliable quality control prior to next‐generation sequencing (NGS).

### 2.5 16S Library preparation and assessment

Samples were shipped to the Georgia Genomics and Bioinformatics Core for library preparation and sequencing. The library preparation process began with normalizing DNA samples to 5 ng/µl. A 5 µl of each sample was used to proceed with the first PCR. The V3‐V4 16S primers with sequencing anchor (S‐D‐Bact‐0341‐b‐S‐17 and S‐D‐Bact‐0785‐a‐A‐21 (Klindworth et al., 2013) were diluted to 2 µM. The first PCR reaction mix consisted of 2.5 µl of each primer, 12.5 µl of KAPA HiFi HotStart ReadyMix, and 2.5 µl of PCR‐grade water (Illumina, 2013). The amplification reaction profile was 95°C–3 min; 15 cycles of 95°C–30 s, 56°C–30 s, and 72°C–30 s; and 72°C–4 min. A post‐PCR cleanup was conducted using 0.8× AMPure beads.

The purified PCR product was resuspended in 50 µl of elution buffer. Next, 5 µl of the first PCR product for each sample was used in the second PCR amplification. The reaction mix consisted of 5 μl first PCR product, 5 µl of each i5 and i5 Unique Illumina indexing primers, 25 µl of KAPA HiFi HotStart ReadyMix, and 10 µl of PCR‐grade water. The amplification reaction profile was 95°C–3 min; 12 cycles of 95°C–30 s, 56°C–30 s, and 72°C–30 s; and 72°C–4 min. The second PCR products were purified using 1× AMPure beads, and the cleaned products were eluted in 25 µl of elution buffer (Illumina, 2013), which constitutes the final 16S sequencing libraries. Library concentrations were measured using the plate fluorometer method.

The molecular weight of the DNA was determined by running the libraries on the Fragment Analyzer. The NGS fragment analysis confirmed the targeted size of 394b for the V3‐V4 region of the 16S amplicon. The libraries were then normalized to have an equal molarity and were pooled together at equal volumes. The concentration of the pool was assessed with Qubit as well as quantitative PCR to have the most accurate reading possible. After this final quality check, the pool was ready for sequencing.

### 2.6 Sequencing

The pool was sequenced on the Miseq PE300 run following the Illumina SOP and recommendations. https://support.illumina.com/documents/documentation/chemistry_documentation/16s/16s-metagenomic-library-prep-guide-15044223-b.pdf

### 2.7 Microbiome analysis

Paired‐end read libraries were checked for quality using fastQC (https://www.bioinformatics.babraham.ac.uk/projects/fastqc). Paired‐end reads were pruned for adaptors and quality using BBDuk (version 37.95) from the BBtools software suite (https://jgi.doe.gov/data-and-tools/bbtools/bb-tools-user-guide/bbmap-guide). Using bowtie2 (Langmead & Salzberg, 2012) (version 2.3.5.1), with default values the paired‐end reads were mapped to chloroplast (KR233156) and mitochondria (KU831325) of *Brassica oleracea*, cultivar C1176, and the cabbage lopper genome (Fu et al., 2018). The unmapped reads to chloroplast, mitochondria, and cabbage lopper were used in the analysis. The 16S amplicon microbiome analysis was carried out using the Qiime2 program (qiime2‐2018‐4; https://docs.qiime2.org; Bolyen et al., 2019). Amplicon sequence variants (ASV) were called using the DADA2 program (‐‐p‐trim‐left‐f 9, ‐‐p‐trim‐left‐r 9, ‐‐p‐trunc‐len‐f 240, ‐‐p‐trunc‐len‐r 240, –p‐n‐reads‐learn 2,000,000) within Qiime2, and chimeric variants were filtered out. ASV classification of nonchimeric sequences were performed using Silva (https://www.arb-silva.de) and Greengenes (http://greengenes.secondgenome.com) with 97% identity datasets using the feature classifier (fit‐classifier‐naive‐bayes) in Qiime2. The sampling depth was set at 9,000. Beta diversity, bacterial diversity between communities, was also calculated using Bray–Curtis in Qiime2. Normalization of ASVs and further microbiome analysis were performed using Calypso (http://cgenome.net/wiki/index.php/Calypso; Zakrzewski et al., 2017). The Ribosomal Database Project's (RDP) Classifier (Wang, Garrity, Tiedje, & Cole, 2007) was used to determine the frequency of ASVs among bacterial taxa, and RDP's Sequence Match utility was used to blast ASV sequences against those of established bacterial type strains.

## 3 RESULTS AND DISCUSSION

**Figure 2:**
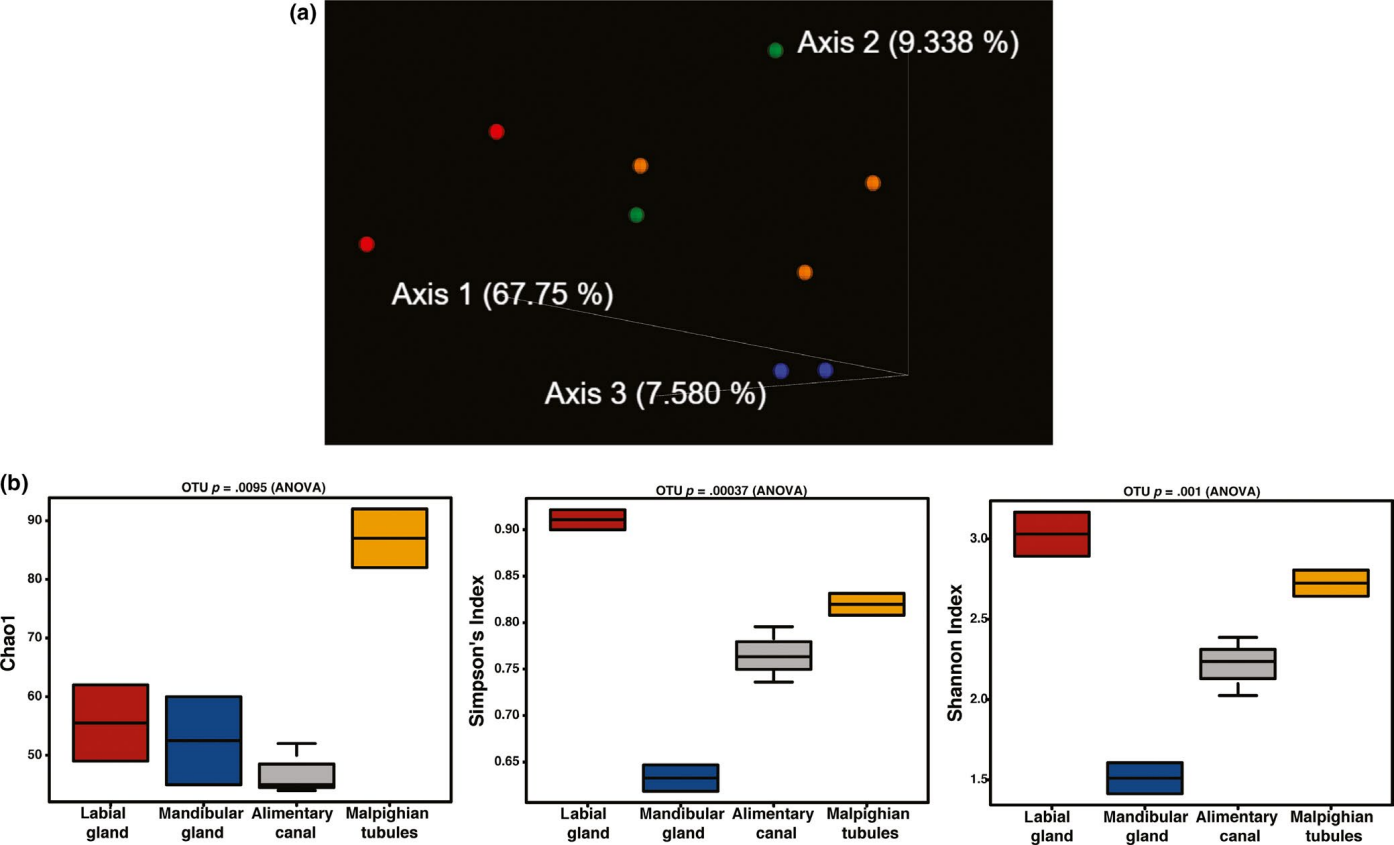
Analysis of Illumina 16S sequencing of *T. ni* organs. (a) Bray–Curtis PCOA emperor plot. Labial gland (Red), mandibular gland (Blue), alimentary canal (Orange), and Malpighian tubules (Green) showed separation among the four tissue types with most the variation in the first axis at 67.75%. (b) Chao1 reflects the greater abundance of low abundance ASVs in the Malpighian tubules. Simpson's and Shannon indices, while weighing more on evenness or richness, respectively, still show similar patterns with labial glands the highest and the mandibular glands the lowest scores

Illumina sequencing of two samples each of Malpighian tubules, mandibular glands, labial glands, and three replicates of alimentary canals resulted in raw reads from a high of 72,647 in labial gland #2 to a low of 20,105 in labial gland #1 (Table 1). The counts were subsequently cleaned of *T. ni* mitochondria, collard chloroplast, and additional *T. ni* sequences, which resulted in minor losses of total raw reads (Table 1). Subsequent filtering, denoising, and merging sequences resulted in final read counts from a high of 32,876 for Malpighian tubule 2 to a low of 9,012 for the alimentary canal 2. Consequently, 9,000 reads were used for the sampling depth. The Bray–Curtis PCOA plot showed separation among the four tissue types (Figure 2a).

**Table 1:** Number of counts for each replicate before and after cleaning

Organ and sample ID	Raw counts	Removed mitochondria, chloroplast	Removed cabbage lopper	Filtered denoised merged	Final nonchimeric
Malpighian tubules—tubule 1	54,120	53,842	50,477	30,343	29,887
Malpighian tubules—tubule 2	49,032	48,964	48,829	33,401	32,876
Mandibular glands—mangland 1	39,035	38,902	38,841	27,520	27,238
Mandibular glands—mangland 2	34,517	34,391	34,378	24,881	24,671
Alimentary canal—midgut 1	29,595	29,528	23,118	9,014	9,012
Alimentary canal—midgut 2	65,469	63,722	55,762	29,606	29,361
Alimentary canal—midgut 3	55,591	55,405	51,009	30,163	30,156
Labial glands—gland 1	20,105	20,056	18,910	9,533	9,447
Labial glands—gland 2	72,647	72,294	66,364	29,276	29,147

Analysis of the scrubbed DNA from the organ samples provided information about abundance and species diversity using the Calypso Diversity utility (http://cgenome.net/wiki/index.php/Calypso). These indices give a broad view of the number of species, or richness and the evenness of the number of individuals in the species (Kim et al., 2017). The number of ASVs detected in each organ is reflected in the alpha diversity plots. The greatest number of ASVs was detected in the Malpighian tubules (Figure 2b) in Chao 1, which adds diversity based on the frequency of singleton and doubleton ASVs. Other measures of species diversity do not incorporate low abundance ASVs (Simpson and Shannon). Shannon is more highly weighted on richness while Simpson on evenness between the species. However, both indices give the highest score to the labial glands and the lowest to the mandibular glands (Figure 2b).

The distribution of identified ASVs among all phyla of bacteria was examined using Ribosomal Database Project's (RDP) Classifier (Wang et al., 2007, see additional data A and B found at figshare: https://doi.org/10.6084/m9.figshare.11387748). Individual ASVs were identified using RDP's Sequence Match utility to blast their sequences against those of established bacterial type strains. Appendix Table A1 contains the species name associated with the ASV. Among ASVs encountered across all organs investigated, the predominant phyla observed (and percentage of detected ASVs) were the Firmicutes (22.8%), Actinobacteria (20.3%), Proteobacteria (37.6%), and Bacteriodetes (11.5%). Two of the ASVs were at high, and similar, abundance across all organs. Their 16S sequences most closely matched *Diaphorobacter nitroreducens* (Betaproteobacteria) and *Cloacibacterium normanense* (Flavobacteriia). Although not nearly as abundant as these two ASVs, other sequences observed across midgut, labial gland, mandibular gland, and Malpighian tubules included those most closely matching type strains of *Acinetobacter baumannii*, *Enterobacter cloacae/Klebsiella pneumoniae*, *Herbaspirillum aquaticum/huttiense*, *Propionibacterium acnes*, and several pseudomonads, representing the Gammaproteobacteria, Betaproteobacteria, and Actinobacteria. The normalized frequencies of the ten most abundant ASVs for each organ, and their levels in the other organs, are shown in Table 2.

**Table 2:** Normalized abundances of the most prevalent ASVs encountered

Nearest sequence(s)	Midgut	Malpighian tubule	Mandibular gland	Labial gland
*Cloacibacterium normanense*	5.41	5.13	6.85	3.28
*Diaphorobacter nitroreducens*	6.12	5.67	6.10	4.07
*Pseudomonas nitroreducens*	0.19	0.51	nd	4.25
*Flavobacterium chungbukense*	nd	0.60	nd	2.82
*Pseudomonas grimontii* and seven others	0.22	0.38	nd	2.04
*Pseudomonas putida Pseudomonas monteilii*	0.33	nd	nd	1.96
*Caulobacter fusiformis*	nd	0.36	nd	1.76
*Acinetobacter baumannii*	1.48	1.11	1.14	0.74
*Pseudomonas poae* and five others	1.41	0.62	0.26	0.68
*Herbaspirillum aquaticum Herbaspirillum huttiense*	1.37	1.19	0.46	0.68
*Anaerococcus octavius*	nd	nd	nd	1.32
*Pseudomonas aeruginosa*	0.71	1.12	1.23	0.32
*Xanthobacter flavus*	nd	nd	nd	1.23
*Propionibacterium acnes*	1.2	1.09	0.78	nd
*Lactobacillus crispatus*	0.34	1.19	0.24	nd
*Methylobacterium radiotolerans*	nd	nd	nd	1.19
*Propionibacterium acnes*	0.59	1.03	0.56	1.08
*Enterobacter cloacae Klebsiella pneumoniae*	1.07	1.04	0.98	0.97
*Pseudomonas salomonii*	1.05	0.64	0.14	0.42
*Pseudomonas salomonii*	0.89	0.46	nd	0.74
*Streptococcus oralis*	0.34	1.00	0.49	nd
*Cloacibacterium normanense*	0.75	0.90	0.94	nd
*Diaphorobacter nitroreducens*	0.52	0.85	0.78	nd
*Enterobacter cloacae*	0.83	0.41	0.78	0.48
*Corynebacterium lactis*	0.53	0.74	0.80	nd

Note

The 10 most abundant ASVs from each organ are included. Bacterial taxa named represent the best matches from among type strains included in the Ribosomal Database Project II, identified using the Sequence Match Utility. Fill colors represent normalized abundance as follows: Red > 3, orange 3–1.75, yellow 1.74–0.75, green 0.74–0.38, and blue < 0.38.

**Figure 3:**
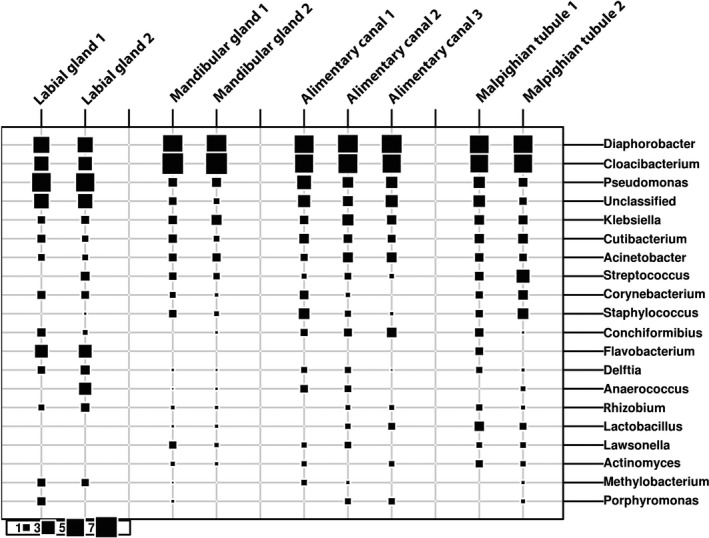
The bubble plot shows the proportion of the ASVs for each genus as a percentage of the whole population of the sample. The size of the squares and their numbers indicate levels of abundance for each genus in each individual replicate. For example, Pseudomonas is highest in the labial glands and lowest in the mandibular glands

**Figure 4:**
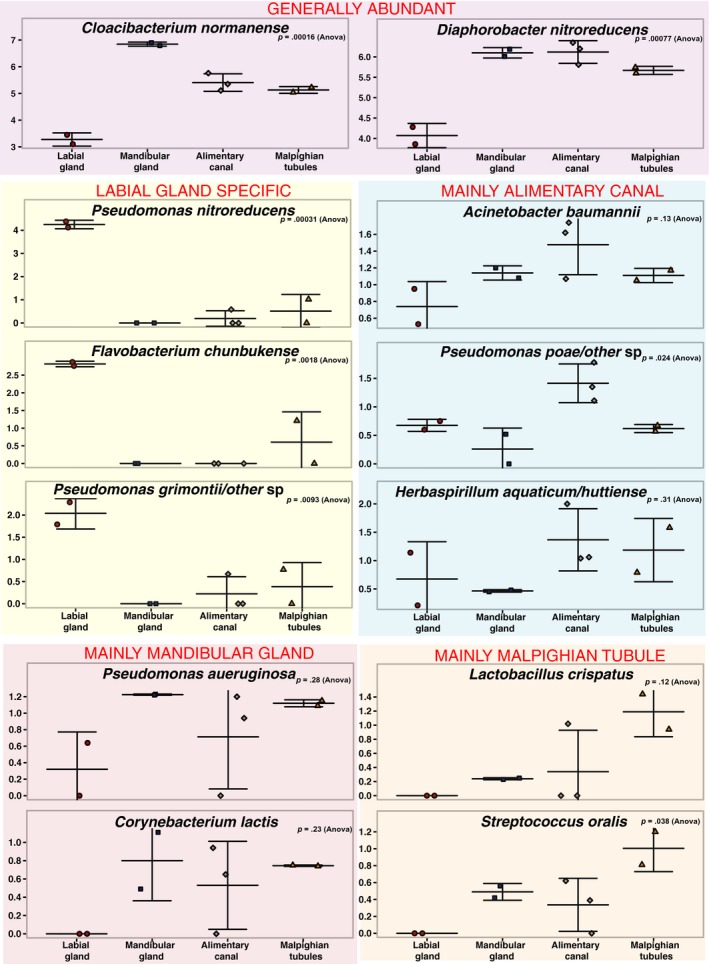
Abundance of selected ASVs in *T. ni* organs. Genus and species epithets are based on the best amplicon sequence match among type strains in the Ribosomal Database Project II

Among the organs examined, the labial glands appeared to have the most distinctive microbiome, possessing several abundant ASVs that were either absent or of low numbers in the other organs, and conversely, lacking ASVs that appeared in the other organs (Table 2). Among those, ASVs that were predominant in the labial glands and not elsewhere are three apparent pseudomonads and a *Flavobacterium* (Figure 3). The sequence of the most abundant of these ASVs matches only that of *Pseudomonas nitroreducens* among type strains in the RDP. The two other distinct pseudomonads appear most closely related to type strains of either *Pseudomonas putida*/*monteilli*, or *Pseudomonas grimontii* (and seven other similar species; Figure 4). The *Flavobacterium* sequence associated with the labial glands appeared most closely related to *Flavobacterium chungbukense*. Several additional abundant ASVs detected in labial glands which were at low or undetectable levels in the other organs best matched type strains of *Caulobacter fusiformis*, *Xanthobacter flavus*, *Methylobacterium radiotolerans, and Anaerococcus octavius* (Table 2); the first three belonging to class Alphaproteobacteria and the last to the Clostridia.

In terms of the distribution of ASVs among phyla and families of bacteria, the alimentary canal bacteria of *T.* *ni* larvae feeding on collards appeared similar to other lepidopteran species (Jones et al., 2019; Paniagua Voirol, Frago, Kaltenpoth, Hilker, & Fatouros, 2018). The two most abundant ASVs from the midgut and other organs, apparently belong to the genera *Cloacibacterium* and *Diaphorobacter*, members of which have been previously reported from insect gut microbiomes (Montagna et al., 2016; Ravenscraft, Kish, Peay, & Boggs, 2019). Interestingly, a *Diaphorobacter* isolate closely related to the ASV from our study was able to utilize the organophosphate insecticide Triazophos as its sole carbon source (Yang et al., 2011), suggesting the possibility that it could contribute to insecticide resistance.

While the microbiomes of the alimentary canal, Malpighian tubules, and mandibular glands appeared quite similar in terms of phylogenetic diversity that of the labial glands appeared to be significantly different. ASVs that appeared to be highly enriched in, or unique to, the labial glands were primarily from among the Proteobacteria, particularly the *Pseudomonas*. Most prominent among these was an ASV whose sequence perfectly matched *Pseudomonas nitroreducens*. In the labial glands, this was the most abundant ASV and was either undetectable or present at much lower levels in the other organs. Huang, Sheng, and Zhang (2012) isolated *Pseudomonas nitroreducens* from the gut of the scarab, *Holotrichia parallela*, and found it to be cellulolytic, suggesting that the ASV found in the current study could contribute to early digestive processes in *T. ni*.

The mandibular and labial glands both open into the oral cavity of the larva, which would seemingly be exposed to the same inoculating population of bacteria. Yet their microbiomes appear to be quite different. This suggests that the conditions within the two organs exert different selective pressures on bacteria attempting to colonize them. The functions of the two glands, and their internal chemistries, have been shown to be significantly different. In addition to silk production, the labial glands of lepidopteran insects have been shown to produce many enzymes and peptides with both digestive and defensive roles (Rivera‐Vega, Galbraith, et al., 2017). While the mandibular glands are less well studied, several species have been shown to produce 2‐acyl‐1,3‐cyclohexanediones, which can serve as larval trail marking pheromones or cuticular hydrocarbons (Fitzgerald, Kelly, Potter, Carpenter, & Rossi, 2015; Howard & Baker, 2004). In addition, many chemosensory and odorant‐binding proteins have been found in mandibular gland secretions, suggesting that the gland is involved in chemical communication (de la Paz Celorio‐Mancera et al., 2012). Whether this is due to a difference in pH, differential production of antimicrobial factors by the two glands, or altogether different elements, remains to be elucidated.

## CONFLICT OF INTEREST

None declared.

## AUTHOR CONTRIBUTION

Susan Lawrence: Conceptualization‐Equal, Project administration‐lead, Writing‐review and editing‐equal. Nicole Novak: Conceptualization‐equal, investigation‐equal, writing‐review & editing‐supporting, Methodology‐lead, Visualization‐equal. Jonathan Shao: Data curation‐Lead, Formal analysis‐lead, writing‐review and editing‐supporting. Saikat Kumar Ghosh: Investigation‐supporting, writing‐review & editing‐supporting. Michael Blackburn: Conceptualization‐equal, Visualization‐equal, writing‐original draft‐lead, writing‐review & editing‐equal.

## ETHICS STATEMENT

None required.

## Data Availability

Raw Illumina MiSeq paired‐end sequence data and processed reads (ASV sequences) are available in the NCBI Sequence Read Archive database under BioProject accession number PRJNA565937 (https://www.ncbi.nlm.nih.gov/bioproject/PRJNA565937). A direct link to the ASV sequences; https://sra-pub-src2.s3.amazonaws.com/SRR10593235/Appendix2_ASV_SRA_clean.fasta.1 Additional data A and B are available in figshare at https://doi.org/10.6084/m9.figshare.11387748: (A) Bacterial taxa associated with the alimentary canal, labial glands, Malpighian tubules, and mandibular glands of cabbage loopers, Trichoplusia ni, fed on collards, *Brassica oleracea*. Numbers represent the percentage of ASVs in the associated taxon; among all, ASVs present in the next higher taxonomic level; (B) ASV numbers and associated taxonomic classifications.

## APPENDIX 1

**Table A1:** ASV # associated of species associated with most abundant species

*Cloacibacterium normanense*	*cfdd1cadee04610eab7db450cb656ec4*
*Diaphorobacter nitroreducens*	*5a788c946dcbb5c3fe47ac3fa484da98*
*Pseudomonas nitroreducens*	*d3e24c6b0117f5219acbb44eed069d0b*
*Flavobacterium chungbukense*	*066e39b3f3225390cf5ef42109770b56*
*Pseudomonas grimontii and seven others*	*ea87601205b0074f7b18dfd9ccb370fe*
*Pseudomonas putida Pseudomonas monteilii*	*bb04555e59b1316dd2e4a7aa2cced428*
*Caulobacter fusiformis*	*bf104f9354e535f2bb7c0ab52ffab767*
*Acinetobacter baumannii*	*81ea51b2243fa31c137605ecad5bf2b5*
*Pseudomonas poae and five others*	*70732d0e5d629c166501b55eac6abbc2*
*Herbaspirillum aquaticum Herbaspirillum huttiense*	*4db032460717fddc6c8f366de7f4587d*
*Anaerococcus octavius*	*da523a955158788b81b3ea200d9fb8fc*
*Pseudomonas aeruginosa*	*66927999ec93cb77b8d2daaa33274f44*
*Xanthobacter flavus*	*0e1417dda57fd734240c92710ac48773*
*Propionibacterium acnes*	*db9e99c2752923ea95298a269a827078*
*Lactobacillus crispatus*	*5b1367fa5e56e3af51bc8685ed0607d4*
*Methylobacterium radiotolerans*	*82d590da46a09ac380af801557b6e8f1*
*Propionibacterium acnes*	*b60c3aee69390c1119f449faf7ec6f52*
*Enterobacter cloacae Klebsiella pneumoniae*	*3dddff824201c8b54a6605c02f4235d0*
*Pseudomonas salomonii*	*1136b8a33d96e279aaf005f259862145*
*Pseudomonas salomonii*	*955ca35c0e8801881201726a11082989*
*Streptococcus oralis*	*76ad4d407f280c66c03285b310085fd4*
*Cloacibacterium normanense*	*58f86aa5b1c89cb9eb6c21b1c6984935*
*Diaphorobacter nitroreducens*	*d90d4b51e4ac09de7d3207a2cedc7b37*
*Enterobacter cloacae*	*9f02f292e6bc958efd0df77e21f21968*
*Corynebacterium lactis*	*d178c8680a7a1ea1f63c39a1f28e6a18*
